# Impact of Outpatient Neuraminidase Inhibitor Treatment in Patients Infected With Influenza A(H1N1)pdm09 at High Risk of Hospitalization: An Individual Participant Data Metaanalysis

**DOI:** 10.1093/cid/cix127

**Published:** 2017-02-12

**Authors:** Sudhir Venkatesan, Puja R. Myles, Jo Leonardi-Bee, Stella G. Muthuri, Malak Al Masri, Nick Andrews, Carlos Bantar, Gal Dubnov-Raz, Patrick Gérardin, Evelyn S. C. Koay, Tze Ping Loh, Ziad Memish, Elizabeth Miller, Maria E. Oliva, Barbara A. Rath, Brunhilde Schweiger, Julian W. Tang, Dat Tran, Tjasa Vidmar, Pauline A. Waight, Jonathan S. Nguyen-Van-Tam

**Affiliations:** 1 Division of Epidemiology and Public Health, University of Nottingham, and; 2 MRC Unit for Lifelong Health and Ageing, University College London, United Kingdom;; 3 Ministry of Health, Riyadh, Kingdom of Saudi Arabia;; 4 Public Health England, United Kingdom;; 5 Department of Infection Control, Hospital San Martín de Paraná, Entre Ríos, Argentina;; 6 Edmond and Lily Safra Children’s Hospital, Sheba Medical Center, Israel;; 7 Pôle Femme Mère Enfant, Centre Hospitalier Universitaire de la Réunion,; 8 Institut National de la Santé et de la Recherche Médical (INSERM) Centre for Clinical Investigation (CIC1410), Centre Hospitalier Universitaire de la Réunion, Saint Pierre,; 9 Unité Mixte 134 PIMIT "Processus Infectieux en Milieu Insulaire Tropical" (Centre National de la Recherche Scientifique 9192, INSERM U1187, Institut Recherche et Développement 249), Université de la Réunion, CYROI "Cyclotron Réunion-océan Indien", Sainte Clotilde, Reunion;; 10 Molecular Diagnostic Centre, Department of Laboratory Medicine National University Hospital, and; 11 Department of Pathology, National University of Singapore;; 12 College of Medicine, Alfaisal University, Riyadh, Kingdom of Saudi Arabia;; 13 Department of Pediatrics, Charité University Medical Center, and; 14 National Reference Centre Influenza at Robert Koch Institute, Berlin, Germany;; 15 University Hospitals Leicester, and; 16 Department of Infection, Immunity and Inflammation, University of Leicester, United Kingdom;; 17 Division of Infectious Diseases, Department of Paediatrics, Hospital for Sick Children, University of Toronto, Canada;; 18 General Hospital Slovenj Gradec, Slovenia

**Keywords:** influenza, neuraminidase inhibitors, individual participant data metaanalyses, hospitalization, pandemic.

## Abstract

**Background.:**

While evidence exists to support the effectiveness of neuraminidase inhibitors (NAIs) in reducing mortality when given to hospitalized patients with A(H1N1)pdm09 virus infection, the impact of outpatient treatment on hospitalization has not been clearly established. We investigated the impact of outpatient NAI treatment on subsequent hospitalization in patients with A(H1N1)pdm09 virus infection.

**Methods.:**

We assembled general community and outpatient data from 9 clinical centers in different countries collected between January 2009 and December 2010. We standardized data from each study center to create a pooled dataset and then used mixed-effects logistic regression modeling to determine the effect of NAI treatment on hospitalization. We adjusted for NAI treatment propensity and preadmission antibiotic use, including “study center” as a random intercept to account for differences in baseline hospitalization rate between centers.

**Results.:**

We included 3376 patients with influenza A(H1N1)pdm09, of whom 3085 (91.4%) had laboratory-confirmed infection. Eight hundred seventy-three patients (25.8%) received outpatient or community-based NAI treatment, 928 of 2395 (38.8%) with available data had dyspnea or respiratory distress, and hospitalizations occurred in 1705 (50.5%). After adjustment for preadmission antibiotics and NAI treatment propensity, preadmission NAI treatment was associated with decreased odds of hospital admission compared to no NAI treatment (adjusted odds ratio, 0.24; 95% confidence interval, 0.20–0.30).

**Conclusions.:**

In a population with confirmed or suspected A(H1N1)pdm09 and at high risk of hospitalization, outpatient or community-based NAI treatment significantly reduced the likelihood of requiring hospital admission. These data suggest that community patients with severe influenza should receive NAI treatment.

## Summary

Our findings suggest that in populations at high risk for hospital admission, patients with laboratory-confirmed or clinically diagnosed A(H1N1)pdm09 infection, NAI treatment in the community or outpatient settings is associated with reduced likelihood of subsequent hospital admission.

Neuraminidase inhibitors (NAIs) were widely used in hospitals, outpatient clinics, and primary care settings during the influenza A(H1N1) pandemic in 2009–2010. Although licensed on the basis of symptom reduction, deployment in 2009–2010 was mainly targeted toward reducing complications, hospital admissions, and deaths. Data from randomized trials pertaining to important public health outcomes, such as reductions in mortality and hospital admissions, for patients with A(H1N1)pdm09 influenza are lacking. In otherwise healthy patients with seasonal influenza, low case severity and low frequency of severe outcomes contribute to a lack of statistical power in randomized studies [1–3]. Notwithstanding, summaries of observational data suggest that NAIs reduced mortality in hospitalized patients during the 2009–2010 pandemic [4, 5]. Likewise, a metaanalysis of observational data on seasonal influenza prior to 2009 also suggests that NAIs given to high-risk community patients with influenza may reduce subsequent hospitalization [6]. To the best of our knowledge, similar data on hospitalization during the 2009–2010 pandemic period are absent. We therefore performed a global individual participant data (IPD) metaanalysis to address this question.

## METHODS

### The PRIDE Research Consortium

Research centers participating in the Post-pandemic Review of anti-Influenza Drug Effectiveness (PRIDE) study were identified while conducting a metaanalysis of published studies on the effectiveness of NAI treatment in hospitalized patients [4]. A detailed description of the PRIDE study has been published previously [5]. In total, the PRIDE consortium obtained data on 170858 potentially eligible patients from 81 research centers in 38 countries across 6 World Health Organization regions. A subset of these centers provided community or outpatient data, which were then used for the current analysis. No data were provided or funded for collection by pharmaceutical companies. The protocol for this study was registered with the PROSPERO register of systematic reviews (CRD42011001273) [7].

### Data Standardization, Exposure, Outcome, and Covariates

Data were available on community patients or those attending outpatient clinics with laboratory-confirmed or clinically diagnosed influenza A(H1N1)pdm09 from 9 centers (Argentina, Canada, France, Germany, Israel, Saudi Arabia, Singapore, Slovenia, and the United Kingdom). These were standardized using a data dictionary (Supplementary Table 1) before pooling for analysis. The primary outcome variable was influenza-related hospital admission as determined by case records linking admission to the influenza illness episode. The primary exposure variable was treatment with an NAI initiated in any community or outpatient setting as compared to no NAI treatment in the community or outpatient setting. If data were available, we further distinguished early treatment (NAI started ≤2 days after symptom onset) vs later treatment (>2 days after symptom onset). We excluded from all our analyses those patients who received NAI treatment in the community on the day of hospital admission, on the grounds that treatment would not have had sufficient opportunity to work in these patients. This exclusion also accounts for any physician decisions to prescribe NAIs taken after a decision to admit the patient to the hospital, amounting to confounding by indication. Covariates adjusted for in the final multivariable model were outpatient or community-based antibiotic treatment (yes/no) and propensity score (by quintile) for receiving NAI treatment in the community.

### Propensity Scoring

We computed propensity scores for the likelihood of community-based NAI treatment for individual patients within each contributing dataset using the method described by Hirano and Imbens [8]. Multivariable logistic regression models developed to calculate propensity scores included the following covariates: age, sex, presence of a comorbid condition (yes/no), and an indicator of disease severity (in order of preference: documented severe respiratory distress or shortness of breath). The resulting propensity scores were then categorized into quintiles for use in subsequent analyses.

### Statistical Analyses

In the primary analysis, we used a mixed-effects logistic regression model to investigate the association between community-based NAI treatment and subsequent hospital admission using the xtmelogit command in Stata (version 14). To account for differences in baseline outcome between individual datasets, we included individual study centers as a random intercept. We ran both unadjusted and adjusted models, the latter containing covariates for community-based antibiotic treatment and propensity scores. To allow for comparisons between the unadjusted and adjusted models, we included missing data as a dummy variable category. The C-statistic (area under the receiver operating characteristic curve) was used to assess model fit. Where data were available, we explored the potential impact of timing of NAI administration (early NAI treatment vs later NAI treatment) on hospitalization. We also performed stratified analysis in patients with laboratory-confirmed A(H1N1)pdm09 influenza and adults (aged ≥16 years) and children.

Furthermore, we carried out an additional analysis restricted to patients with high-risk conditions. Patients were classified as having a high-risk condition if they had at least 1 chronic illness recorded that would trigger seasonal influenza vaccination [9] or were aged ≥65 years.

Results from our mixed-effects logistic regression models are presented as odds ratios (ORs) with 95% confidence intervals (95% CIs).

## RESULTS

**Figure 1.**
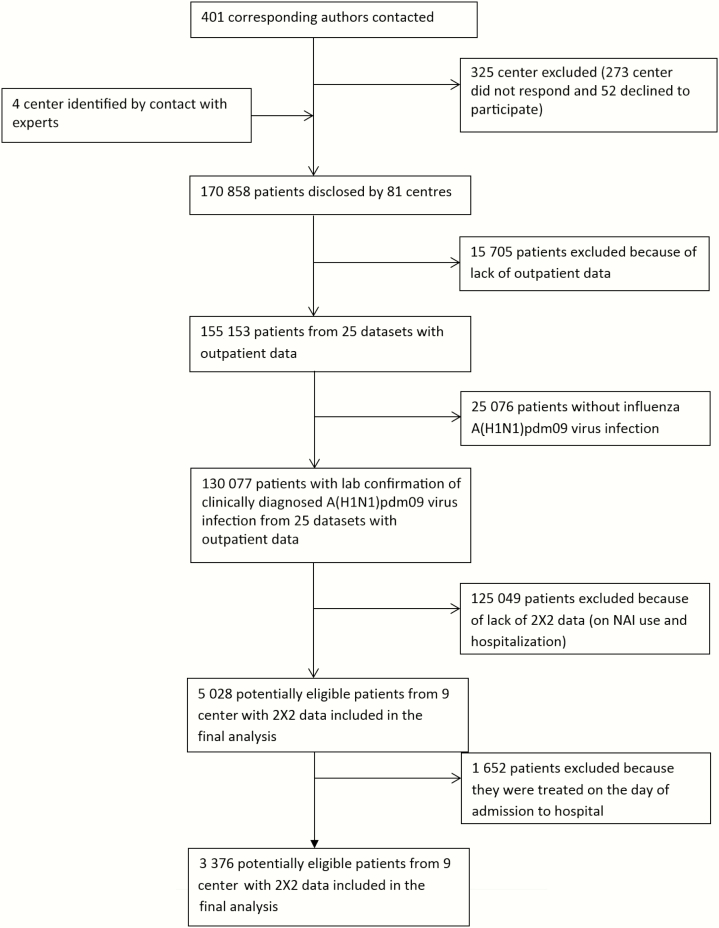
Study flow diagram. Abbreviation: NAI, neuraminidase inhibitor.

We received outpatient data on 130077 patients with clinically or laboratory-confirmed influenza from 25 centers. However, 16 centers (n = 125049 patients) offered surveillance data that did not contain clinical data on either NAI use or subsequent hospitalization status. Therefore, the final study population comprised 3376 patients from 9 study centers (Figure 1). Data from 7 of the 9 included study centers (n = 1183 patients) came from outpatient (ambulatory care) clinics attached to hospitals. Of the remaining 2 study centers, 1 provided community surveillance data collected by a local Ministry of Health (n = 1762) and the other provided data from primary care (n = 431). Three of the 9 included centers (Canada, Germany, and Israel; total n = 535) were from pediatric outpatient clinics and comprised entirely of patients aged <18 years. Patients from the German study center were particularly young with a median age of 1.4 years (Supplementary Table 3).

Of 3376 patients in our pooled dataset, 3085 (91.4%) had laboratory-confirmed A(H1N1)pdm09 infection; 1747 (51.8%) were children (<16 years) and 67 (2.0%) were aged >64 years. Overall, 1705 (50.1%) were admitted to the hospital and 928 (928/2395; 38.7%) had clinically observed shortness of breath or respiratory distress as a marker of severity. Where data were available (n = 473), the median interval between date of symptom onset and date of NAI treatment initiation was 1 day (interquartile range [IQR], 0–3) for the whole study population, 1 day (IQR, 0–2) for nonhospitalized patients, and 1 day (IQR, 0–4) for hospitalized patients. Of the hospitalized patients, where calculable (n = 1363), the median interval between date of symptom onset and date of hospital admission was 2 days (IQR, 1–3). General characteristics of the study population are presented in Table 1. About one half (50.1%) of the cohort was eventually admitted to the hospital, and 38.8% had 1 or more indications of severe respiratory illness as denoted by observed shortness of breath or respiratory distress. In addition, fewer than 2% of the cohort were elderly and about 72% had no recorded comorbid conditions, suggesting that most patients were young and previously healthy. Indeed, 51.8% were children and 37.1% of women aged 15–44 years were pregnant, reflecting the inclusion of 1 obstetric clinic (n = 81) within the data.

**Table 1.** General Characteristics of the Study Population

Variable	All Patients (n = 3376)	Nonhospitalized (n = 1671)	Hospitalized (n = 1705)
Number of male cases	1712 (50.71)	859 (51.41)	853 (50.03)
Age, median (interquartile range) in years (n = 3253)	14 (4.95 to 27.88)	14 (6.24 to 27)	15 (3.85 to 28.47)
Population group (no. of persons)			
Adults (≥16 years)	1506 (44.61)	730 (43.69)	776 (45.51)
Children (<16 years)	1747 (51.75)	879 (52.60)	868 (50.91)
Aged ≥65 years	67 (1.98)	22 (1.32)	45 (2.63)
Pregnant women^a^ (n = 741)	237/639 (37.09)	121/278 (43.53)	116/361 (32.13)
Country			
Argentina	17 (0.50)	13 (0.78)	4 (0.23)
Canada	148 (4.34)	113 (6.76)	35 (2.05)
France	81 (2.40)	55 (3.29)	26 (1.52)
Germany	314 (9.29)	161 (9.63)	153 (8.95)
Israel	73 (2.16)	36 (2.15)	37 (2.16)
Saudi Arabia	1762 (52.11)	613 (36.68)	1,149 (67.19)
Singapore	490 (14.49)	242 (14.48)	248 (14.50)
Slovenia	60 (1.77)	24 (1.44)	36 (2.11)
United Kingdom	431 (12.77)	414 (24.78)	17 (1.00)
A(H1N1)pdm09 diagnosis			
Laboratory confirmed	3,085 (91.38)	1,522 (91.08)	1,563 (91.67)
Clinically diagnosed	291 (8.61)	149 (8.92)	142 (8.33)
Severe disease (n = 2395) (severe respiratory distress or shortness of breath at presentation)	928/2395 (38.75)	321/991 (32.39)	607/1404 (43.23)
Comorbidity			
Any comorbidity (n = 2945)	824/2945 (27.98)	302/1,257 (24.03)	522/1688 (30.92)
Asthma (n = 1172)	214/1172 (18.26)	91/634 (14.35)	123/538 (22.86)
Chronic obstructive pulmonary disease (n = 902)	120/902 (13.30)	38/471 (8.07)	82/431 (19.03)
Other chronic lung disease (n = 2257)	290/2257 (12.85)	98/871 (11.25)	192/1386 (13.85)
Heart disease (n = 614)	20/614 (3.26)	4/294 (1.36)	16/320 (5.00)
Renal disease (n = 2299)	92/2299 (4.00)	40/879 (4.55)	52/1420 (3.66)
Liver disease (n = 541)	11/541 (2.03)	5/258 (1.94)	6/283 (2.12)
Cerebrovascular disease (n = 490)	7/490 (1.43)	1/242 (0.41)	6/248 (2.42)
Neurological disease (n = 2448)	57/2488 (2.33)	9/963 (0.93)	48/1485 (3.23)
Diabetes (n = 2449)	135/2449 (5.51)	47/964 (4.88)	88/1485 (5.93)
Immunosuppression (n = 2390)	97/2390 (4.06)	36/918 (3.92)	61/1472 (4.14)
Community/outpatient NAI treatment			
Any NAI treatment	873 (25.82)	653 (39.08)	220 (12.90)
Oseltamivir ^b^ (n = 2945)	590 /2,94 (20.03)	385/1257 (30.63)	205/1688 (12.14)

Study size n = 3376. Percentages presented are column percentages unless other denominators are specified.

Abbreviation: NAI, neuraminidase inhibitor.

^a^Proportions were calculated as a percentage of pregnant patients among female patients of reproductive age; the broader age range was selected in preference to the World Health Organization definition (15–44 years) after consultation with data contributors to reflect the actual fertility experience of the sample. This includes data from an obstetric outpatients clinic (n = 81).

^b^Where it was explicitly stated that the NAI administered was oseltamivir.

Of the 1705 patients who were hospitalized, we had data on the subsequent course in 1433 patients. Of these hospitalized patients, 1155 (80.6%) were treated with NAIs in-hospital, 147 patients (10.3%) were subsequently admitted to critical care facilities (of which 119 [80.9%] treated, 28 [19.1%] untreated), and 14 patients (1%) died (13 [92.9%] treated, 1 [7.1%] untreated). In a smaller subgroup of 1392 patients on whom we had data relating to pneumonia, 215 (15.5%) were found to have clinical signs of pneumonia, with radiographic confirmation of pneumonia in 101 (7.3%) patients (64 [63.4%] treated, 37 [36.6%] untreated). Further, we had data on length of subsequent hospital stay in a smaller subgroup of 522 patients where the median length of stay was 3 days (IQR, 2–5; median, 3 days [IQR, 2–5] in treated and median, 2 days [IQR, 1.5–5] untreated). Of the 1647 patients who were excluded from the analyses because they were hospitalized on the same day as NAI treatment initiation, 116 (7%) were admitted to critical care facilities and 23 (1.4%) died. In 1595 of these patients on whom we had pneumonia data, 154 (9.7%) were found to have had clinical signs of pneumonia; in 31 (1.9%) of these patients the pneumonia was radiologically confirmed. The median length of hospital stay in an even smaller subgroup (n = 186 where data were available) was found to be 3 days (IQR, 2–5).

In patients with laboratory-confirmed or clinically diagnosed A(H1N1)pdm09 influenza, after adjustment for community-based antibiotic treatment and propensity score, the likelihood of hospital admission in patients with outpatient or community-based NAI treatment was 0.24 (95% CI, 0.20–0.30) when compared to no NAI treatment in the community (Table 2). A C-statistic of 0.813 (95% CI, 0.799–0.827) suggested that the predictive performance of our model was acceptable.

**Table 2.** Association Between Neuraminidase Inhibitor Administration and Hospital Admission

Population	Unadjusted Analysis		Adjusted Analysis^a^	
	Unadjusted OR (95% CI)	*P* Value	Adjusted OR (95% CI)	*P* Value
Patients with laboratory-confirmed or clinically diagnosed A(H1N1)pdm09 influenza (n = 3376)	0.23 (0.19 to 0.28)	<.001	0.24 (0.20 to 0.30)	<.001
Patients with laboratory-confirmed A(H1N1)pdm09 influenza (n = 3085)	0.23 (0.19 to 0.28)	<.001	0.24 (0.19 to 0.29)	<.001
Adults (aged ≥16 years) (n = 1506)	0.26 (0.19 to 0.35)	<.001	0.26 (0.19 to 0.35)	<.001
Children (aged <16 years) (n = 1747)	0.22 (0.17 to 0.30)	<.001	0.25 (0.18 to 0.34)	<.001
Patients with at least 1 high-risk condition (n = 1019)	0.26 (0.19 to 0.37)	<.001	0.27 (0.19 to 0.38)	<.001
Early neuraminidase inhibitor treatment (≤2 days after onset) vs later (>2 days) in patients with laboratory-confirmed or clinically diagnosed A(H1N1)pdm09 influenza (n = 473)	0.51 (0.28 to 0.93)	.031	0.44 (0.23 to 0.86)	.016

^a^Adjusted for treatment propensity (by quintile) and community-based antibiotic use.

Abbreviations: CI, confidence interval; OR, odds ratio.

When restricted to laboratory-confirmed A(H1N1)pdm09 patients, the estimate was very similar to the estimate for the overall study population (Table 2). NAI treatment, when compared to no treatment, was associated with reduced odds of hospitalization in both children (adjusted OR, 0.25; 95% CI, 0.18–0.34) and adults (adjusted OR, 0.26; 95% CI, 0.19–0.35). In the subgroup of 473 patients in whom data on the interval between symptom onset and start of NAI treatment were available, early NAI treatment was associated with an adjusted OR of 0.44 (95% CI, 0.23–0.86) when compared to later NAI treatment.

In the pooled dataset, 1019 patients (30.1%) were recorded to have at least 1 high-risk condition. In this subpopulation of higher-risk patients, we also observed a reduction in the odds of hospital admission (OR, 0.27; 95% CI, 0.19–0.38) in those treated with NAIs in the community compared with no NAI treatment. Full results of the sensitivity and stratified analyses are summarized in Table 2.

The hospital admission rate varied widely among each of the 9 included study centers, ranging from 3.94% to 69.3%. To separate any effects that hospital admission rates among centers may have had on the association between NAIs and hospitalization, we did a post hoc stratified analysis by median hospitalization rate (50.6%). After adjusting for community-based antibiotic treatment and propensity score, the pooled OR for the association between NAI treatment and subsequent hospitalization in study centers with a hospital admission rate <50.6% (n = 991) was 1.00 (95% CI, 0.61–1.64), whereas in centers where the admission rate was >50.6% (n = 2385), the OR was 0.17 (95% CI, 0.14–0.22).

## DISCUSSION

In this study, we assembled data from a large cohort of community-based patients who had pandemic influenza in 2009–2010, of whom 91% had laboratory confirmation of influenza A(H1N1)pdm09 infection. The demographic and clinical findings (Table 1) reveal that most patients were young and previously healthy, yet with relatively severe influenza (indicated by the presence of either documented severe respiratory distress or shortness of breath at presentation). As such, we recognize that our results are not generalizable to a wider range of community-based patients with mild pandemic influenza and may not be generalizable to the elderly.

Our main findings (Table 2) suggest that NAI treatment in the community for patients with severe pandemic influenza substantially reduced the likelihood of hospital admission due to influenza A(H1N1)pdm09. In a pandemic context, individuals generally have little or no preexisting cross-reactive immunity to the infecting virus; therefore, effect size might be lower for seasonal (interpandemic) influenza, and our findings should be interpreted with more caution in that context. In a sensitivity analysis restricted to patients with laboratory-confirmed A(H1N1)pdm09, this finding was unaltered; and in patients with underlying at-risk conditions, the risk reduction was greater. A limitation of our analysis is that we did not have data on body mass index and therefore could not include obesity as a high-risk condition.

We also explored potential differences in effect size between adults and children and found that the effect of NAIs in reducing the likelihood of hospital admission was maintained and broadly consistent in both age groups. These findings contrast with our previous analysis of mortality data, in which we failed to demonstrate significantly reduced mortality in hospitalized children treated with NAIs [5]. This discrepancy is potentially explained by the relatively high attack [10] and hospitalization rates in children with A(H1N1)pdm09 [11] compared with a relatively low case fatality rate [12] but could also relate to statistical underpowering in the mortality study [5].

A question of considerable clinical relevance relates to the timing of antiviral treatment in relation to the magnitude of benefit obtained, especially since data already exist to suggest that symptom alleviation and mortality reduction are both diminished by delayed treatment [5, 13]. We were able to perform a sensitivity analysis on 473 NAI-treated patients in whom we had specific data on the timing of symptom onset and antiviral treatment. This revealed that earlier treatment (within 48 hours of symptom onset) was significantly more beneficial than later treatment.

Because the dataset contained so few elderly patients, perhaps reflecting the low incidence of A(H1N1)pdm09 infection in the elderly [14, 15], we were unable to cast any further light on the effectiveness of NAIs in this particular subgroup of patients.

Although smaller than our previous IPD analysis that focused on mortality reduction in hospitalized patients [5], one strength of this study is the relatively large number of patients included from 9 geographically diverse clinical centers. Although we were unable to adjust specifically for disease severity in our multilevel models because of the heterogeneity of severity measures used across individual datasets, we nevertheless included physician-recorded breathlessness and severe respiratory distress when deriving propensity scores. However, we acknowledge that confounding by indication [16] may still be present. If it is, we surmise that physicians may have been more likely to treat severe cases than milder ones or to treat putative at-risk groups such as pregnant women with NAIs. Therefore, the treated group would have a higher underlying likelihood of being admitted to the hospital; this in turn would produce a bias in the analysis, tending toward underestimation of any treatment effect. Likewise, we recognize that some NAI treatment may have been given immediately prior to hospital admission when there was no practical window in which an antiviral drug could have had time to work or perhaps even when the physician had already decided that the patient needed to be admitted. Therefore, we think there is sound clinical rationale for excluding patients in whom NAI treatment was initiated on the day of hospital admission.

Another potential limitation of our propensity scoring approach is that we lacked data on vaccination, albeit knowledge of vaccination status might be associated with physicians’ decisions to prescribe NAIs and to hospitalize patients. To explore this further, we determined that patients whose illness onset was on or before 15 October 2009 could not have been vaccinated due to nonavailability prior to this date. Even if they had been vaccinated, they would not have had time to seroconvert. We subsequently performed a stratified analysis around this date by dividing the overall pooled dataset into “early pandemic” and “later pandemic.” We had onset dates in 2175 patients of whom 903 were on or before 15 October 2009. The adjusted ORs (95% CIs) in both groups were very similar (early pandemic group, 0.12 (0.06–0.24) and late pandemic group, 0.11 (0.07–0.18)]. On this basis, we believe that vaccination is unlikely to have been a major confounder in our study. Other residual confounding is nevertheless possible as these are observational data.

Two obvious drawbacks in our data are the overall high rate of hospitalization in the cohort studied, which limits generalizability to patients at high risk of hospitalization, and the substantial variability between hospitalization rates across individual centers, ranging from 4% to 70%. The dataset with the lowest hospitalization rate was from a surveillance system in the United Kingdom where a national policy was in place for 2009–2010 to offer NAI treatment to all patients with clinically apparent influenza, irrespective of severity. Stratifying the analysis around median hospitalization rate revealed no effect of NAIs in centers below the median but revealed a strong positive effect in centers above the median. We surmise that these data confirm the beneficial effect of NAIs (beyond symptom relief) in patients who are severely unwell and at high risk of hospitalization vs those with milder illness.

To our knowledge, this is the first individual participant data metaanalysis to investigate the association between preadmission NAI antiviral use and hospitalization relating to the 2009–2010 influenza pandemic. As such, these data have potential importance for future pandemic stockpiling and treatment policies and may be of relevance to seasonal epidemics, especially for community patients with relatively severe influenza and those with underlying comorbidities. We note that our point estimates of treatment effectiveness are somewhat higher than the 25% reduction in hospitalization for the treatment of seasonal influenza previously reported by Hsu and colleagues [6]. However, the disparity in effect size might be explained by the fact that the 4 studies [17] metaanalyzed by Hsu et al included patients with generally milder influenza than in the present study. In addition, 3 of these 4 studies were based on diagnoses of influenza-like illness (ILI) without laboratory confirmation and therefore were highly vulnerable to misclassification bias. An earlier pooled analysis of clinical trial data from patients with laboratory-confirmed seasonal influenza also showed a 59% reduction in hospitalization [18]. This was confirmed in recent IPD analysis of seasonal influenza patients that reported a risk reduction of 63% in treated patients (intention-to-treat infected population) [19], which is somewhat similar to our own data. Another study not included in Hsu’s metaanalysis also suggested a 29% reduction in hospitalization associated with oseltamivir but was again based on diagnoses of ILI without laboratory confirmation [20]. A recent study from British Columbia, based on clinically diagnosed cases of pandemic influenza A(H1N1)pdm09, also noted 16% effectiveness of NAIs in reducing hospitalization [21]; however, the hospitalization rate in this cohort was 0.6%, suggesting cases were comparatively very mild. We therefore recognize that our findings reflect the experience of NAI use in a cohort of community patients at high risk of hospitalization. In addition, we noted higher effectiveness in patients with 1 or more comorbidities that would have placed them in a target group for annual seasonal influenza vaccination.

Placed in the context of the limited previous work on this subject, our findings suggest that the greatest benefit from community use of NAIs is likely to be achieved by targeting individuals for treatment who have clinically suspected or proven influenza and who are also in a recognized at-risk group or clinically assessed to have severe influenza (irrespective of comorbid status). In these 2 groups of patients, substantial reductions in the likelihood of hospitalization can be achieved, especially if treatment is commenced within 48 hours of symptom onset. Our data support current advice on NAI treatment given by major public health agencies [22, 23] and the findings of a recent independent report from the UK Academy of Medical Sciences and the Wellcome Trust, which recommend against use of NAIs in the community for the treatment of mild influenza, but advise that patients with severe influenza should be treated as soon as possible [24].

## Supplementary Data

Supplementary materials are available at *Clinical Infectious Diseases* online. Consisting of data provided by the authors to benefit the reader, the posted materials are not copyedited and are the sole responsibility of the authors, so questions or comments should be addressed to the corresponding author.

## Supplementary Material

Supplementary_tables_v2

## Notes

***Author contributions.*** J. S. N.-V.-T., P. R. M., J. L.-B., S. V., and S. G. M. conceived and designed the study. All authors, apart from S. V., J. L.-B., and S. G. M., contributed to the acquisition and local preparation of constituent datasets. S. V., P. R. M., J. L.-B., and S. G. M. contributed to data set amalgamation and standardization, design of statistical analyses, and data analysis. J.S .N.-V.-T., P. R. M., J. L.-B., and S. V. interpreted the data and wrote the paper. All authors contributed to critical examination of the paper for important intellectual content and approval of the final report. Each author acted as the guarantor of data from their individual study center. S. V. had full access to the pooled dataset in the study and takes responsibility for the accuracy of the data analysis. J. S. N.-V.-T. acted as overall guarantor of the manuscript.

**Financial support.** The PRIDE study is funded via an unrestricted educational grant from F. Hoffmann-La Roche, Switzerland (the manufacturers of oseltamivir [Tamiflu]). The funder had no role in protocol design, no opportunity to comment on it, and no opportunity to see it other than via the PROSPERO website; no access to any data (and no rights to future access); no role in analysis or interpretation; no opportunity to preview results/findings before entry into the public domain; and no opportunity to contribute to, preview, or comment on manuscripts and presentations arising from this work. The research contract between the University of Nottingham and the funder is freely available for inspection (commercial details redacted) at: http://www.nottingham.ac.uk/research/groups/healthprotection/projects/pride.aspx

***Potential conflicts of interest.*** B. A. R. reports grants from F. Hoffmann-La Roche to her institution (Charité Universitätsmedizin Berlin) outside the submitted work. D. T. reports grants from the Canadian Institutes of Health Research/SickKids Foundation New Investigator (XG08-049R), the Canadian Institutes of Health Research Catalyst (CAT86860), and the University of Toronto Dean’s Fund Pilot Study Grant during the conduct of the study. J. S. N.-V.-T. reports that a grant to the University of Nottingham from F. Hoffmann-La Roche funded the current study; he also reports grants to the University of Nottingham from GlaxoSmithKline for research in the area of influenza; and nonfinancial support from the European Scientific Working Group on Influenza to lecture on influenza outside the submitted work. All other authors: No potential conflicts of interest. All authors have submitted the ICMJE Form for Disclosure of Potential Conflicts of Interest. Conflicts that the editors consider relevant to the content of the manuscript have been disclosed.
