# Supplementary material for: Impact of Outpatient Neuraminidase Inhibitor Treatment in Patients Infected With Influenza A(H1N1)pdm09 at High Risk of Hospitalization: An Individual Participant Data Metaanalysis
Source: Clin Infect Dis. 2017 Feb 12;64(10):1328–34. doi: 10.1093/cid/cix127 (PMC5411393; doi:10.1093/cid/cix127)
Supplement: Supplementary_tables_v2 [file cix127_suppl_Supplementary_tables_v2.pdf]

Supplementary Table 1: Standardised dataset: data dictionary

Variable name in Stata	Description	Coding
**About the data**		
study_group_id	Study group identifier	Auto-numbering (_n); string
patid	Study specific patient ID as provided by study groups; this will only be retained in the individual datasets but dropped from the pooled dataset	string
auto_patid	Auto-numbered patient ID generated for each study group dataset; this will only be retained in the individual datasets but dropped from the pooled dataset	Auto-numbering (_n)
pride_patid	Unique patient identifier created by concatenation of study_group_id and auto-numbered patient id (auto_patid)	string
country	Country identifier	string
**Patient characteristics**		
age_years	Age in years; continuous variable	Place all under 1s in one category (replace <1s as '0')
sex	gender	1=male<br>0=female
pregnant	Pregnancy<br>(for sub-group analyses including pregnant women, only women of child-bearing age (15-54 years) will be considered)	1= yes<br>0= no<br>(no separate code for 'not applicable'; men and women not of child-bearing age will be dropped from any analyses including pregnant women)
comorbidity	Whether any co-existing comorbidity was present (either as recorded or derived on the basis of a record of one of the following comorbidities: asthma, COPD, other chronic lung disease, heart disease, cerebrovascular disease (not including uncomplicated hypertension), chronic liver disease, chronic renal disease, diabetes, neurological disease (including neurodevelopmental disorders) and immunosuppression)<br>Note: pregnancy will not be considered a 'comorbidity'	1= any comorbidity<br>0= no recorded comorbidity
asthma	Asthma as recorded	1= yes

Variable name in Stata	Description	Coding
		0= no
copd	COPD as recorded	1= yes<br>0= no
other_lung_ds	Chronic pulmonary diseases (other than asthma or COPD)	1= yes<br>0= no
heart_ds	Chronic heart disease as recorded (includes congenital heart disease, hypertension with cardiac complications, chronic heart failure, individuals requiring regular medication and/or follow-up for ischaemic heart disease)	1= yes<br>0= no
renal_ds	Chronic kidney disease (CKD) as recorded including CKD at stage 3, 4 or 5, chronic kidney failure, nephrotic syndrome, kidney transplantation.	1= yes<br>0= no
liver_ds	Chronic liver disease as recorded (including cirrhosis, biliary artesia, chronic hepatitis)	1= yes<br>0= no
cerebrovascular_ds	Cerebrovascular disease as recorded (including stroke, transient ischaemic attack but not including uncomplicated hypertension)	1= yes<br>0= no
neurological_ds	Neurological disease as recorded (including neurodevelopmental disorders)	1= yes<br>0= no
diabetes	Diabetes as recorded (including Type 1 diabetes, type 2 diabetes requiring insulin or oral hypoglycaemic drugs, diet controlled diabetes.)	1= yes<br>0= no
immunosuppression	Immunosuppression due to disease or treatment. Includes patients undergoing chemotherapy leading to immunosuppression; asplenia or splenic dysfunction and HIV infection at all stages.	1=yes<br>0= no
**clinical characteristics**		
onset_date	Date of onset of influenza like illness (ILI) (as recorded in clinical notes)	Recorded as date dd/mm/yy

Variable name in Stata	Description	Coding
admission_date	Date of first admission to hospital (whether to a general ward, HDU or ICU)	Recorded as date dd/mm/yy
time_to_admission	Time (in days) from onset of symptoms to hospital admission	Recorded as number of days
shortness_of_breath	Shortness of breath at presentation (as recorded in clinical notes)	1= yes<br>0= no
severe_respiratory_distress	Severe respiratory distress at admission (as recorded in clinical notes)	1= yes<br>0= no
flu_diag	Method of diagnosis of swine flu whether clinical or laboratory diagnosis (this includes RT-PCR)	1= clinical<br>2= laboratory diagnosis (not otherwise specified) or RT-PCR confirmed
**treatment**		
preadmit_antiviral	NAI given in the community/outpatient clinics (either oseltamivir or zanamivir)	1= yes<br>0= no
preadmit_oseltamivir	Oseltamivir given in the community/outpatient clinics	1= yes<br>0= no
preadmit_antibiotic	Antibiotic given in the community/outpatient clinics	1= yes<br>0= no
oseltamivir_anytime	Oseltamivir administered at any time (whether in the community or in hospital)	1= yes<br>0= no
preadmit_steroid	Steroids given in the community/outpatient clinics	1= yes<br>0= no
**Outcomes**		
Hospitalisation	Admission to hospital (as recorded)	1= yes<br>0=no
death	Death (as recorded)	1= yes<br>0= no
p	Propensity scores for treatment- yes vs. no	Recorded as a continuous variable between 0 and 1
ps_quintile	Propensity scores categorised into quintiles for each individual study for NAI treatment- yes vs. no	Categorical variable with values from 1 to 5 (1=lowest quintile and 5=highest quintile)
p2	Propensity scores for NAI treatment- Early treatment (≤2days) vs. Late treatment (>2 days)	Recorded as a continuous variable between 0 and 1
ps2_quintile	Propensity scores categorised into quintiles for each individual study for early treatment (≤2days) vs. late treatment (>2 days)	Categorical variable with values from 1 to 5 (1=lowest quintile and 5=highest quintile)

Supplementary Table 2: General and clinical characteristics by NAI treatment status

**Variable**	**Treated with NAI** (n=873)	**Not treated with NAI** (n=2,503)
**Number of male cases** (n=1,712)	410 (47.13)	1,302 (52.02)
**Age: median (IQR) in years**	16 (7 to 29)	13.3 (4 to 27)
**Population groups (no. of persons)**		
Adults (≥16 years)	418 (47.88)	1,088 (43.47)
Children (<16 years)	409 (46.85)	1,338 (53.46)
Aged≥ 65 years	17 (1.95)	50 (1.99)
Pregnant women (n=741)*†	86/163 (52.76)	151/476 (31.72)
**A(H1N1)pdm09 diagnosis**		
Laboratory confirmed	864 (98.97)	2,221 (88.73)
Clinically diagnosed	9 (1.03)	282 (11.27)
**Severe disease** (n=2,395) (Severe respiratory distress or shortness of breath)	234/594 (39.39)	694/1,801 (38.53)
**Comorbidities**		
Any comorbidity (n=2,945)	168/616 (27.27)	656/2,329 (28.17)
Asthma (n=1,172)	35/170 (20.59)	179/1,002 (17.86)
COPD (n=902)	3/82 (3.66)	117/820 (14.27)
Other chronic lung disease (n=2,257)	64/566 (11.31)	226/1,691 (13.36)
Heart disease (n=614)	2/69 (2.90)	18/595 (3.30)
Renal disease (n=2,299)	30/503 (5.96)	62/1,796 (3.45)
Liver disease (n=541)	0/22 (0)	11/519 (2.12)
Cerebrovascular disease (n=490)	0/13 (0)	7/490 (1.43)
Neurological disease (n=2,448)	17/568 (2.99)	40/1,880 (2.13)
Diabetes (n=2,449)	26/568 (4.58)	109/1,881 (5.79)
Immunosuppression (n=2,390)	25/515 (4.85)	72/1,875 (3.84)
Hospitalization	220 (25.20)	1,485 (59.33)

Percentages presented in this table are column percentages unless other denominators are specified
† Proportions were calculated as a percentage of pregnant patients among female patients of reproductive age; the broader age range was selected in preference to the WHO definition (15–44 years) after consultation with data contributors to reflect the actual fertility experience of the sample. This includes data from an obstetric outpatients clinic (n=81).
* Where it was explicitly stated that the NAI administered was oseltamivir.

Centre No.	Country	Patients source	Total No. of cases supplied	No. of cases used	Median age, years (Range)	Male (%)	No. of cases with any high-risk condition§ (n=1,074) (%)	No. treated with NAI (Outpatient/ community) (%)	No. with early NAI treatment (≤2 days of symptom onset) (%)*	Hospitalization (%)	Outcomes in those hospitalized (n=1,433)† (%)			Reference‡
											Radiologic pneumonia	Critical care	Death	
11	Germany	Hospital outpatients ; Single Centre	315	**314**	1.4 (<1 to 18)	182 (58.0)	123 (39.2)	13 (4.1)	1 (7.1)	153 (48.7)	51 (16.2)	11 (3.5)	1 (0.3)	-
15	Argentina	Hospital outpatients ; Single Centre	36	**17**	34 (21 to 83)	11 (64.7)	2 (11.8)	3 (17.7)	0 (0)	4 (23.5)	2 (11.8)	1 (5.9)	2 (14.3)	1
18	Canada	Hospital outpatients ; Single Centre	148	**148**	7.6 (<1 to 18)	90 (60.8)	29 (19.6)	32 (21.6)	-	35 (23.7)	15 (10.1)	7 (4.7)	0 (0)	2
25	Israel	Hospital outpatients ; Single Centre	73	**73**	9.1 (1 to 17)	42 (57.5)	30 (41.1)	47 (64.4)	-	37 (50.7)	9 (12.3)	2 (2.7)	0 (0)	3
31	Singapore	Hospital outpatients ; Single Centre	584	**490**	19.1 (<1 to 87.6)	262 (53.5)	187 (38.2)	13 (2.7)	11 (84.6)	248 (50.6)	15 (3.1)	13 (2.7)	3 (0.6)	4
44	France	Hospital outpatients ; Single Centre	549	**81**	24 (13 to 38)	0 (0)	81 (100)	53 (65.4)	46 (86.8)	26 (32.1)	-	0 (0)	0 (0)	5
56	Slovenia	Hospital outpatients ; Single Centre	78	**60**	20.85 (<1 to 78)	35 (58.3)	19 (31.7)	9 (15.0)	5 (71.4)	54 (69.23)	9 (15)	1 (1.7)	3 (5)	-
72	Saudi Arabia	Hospital outpatients & community patients; Surveillance	3,228	**1,762**	17 (<1 to 111)	896 (51.0)	543 (30.82)	446 (25.3)	90 (63.8)	1,149 (65.2)	-	112 (6.4)	5 (0.3)	-
80	UK	Hospital patients & community patients; Hospitalised	2,741	**431**	15 (<1 to 75.4)	194 (45.01)	5 (1.1)	257 (59.6)	196 (76.3)	17 (3.94)	-	-	-	6
TOTAL (9 centres)				**3,376**	14 (<1 to 111)	1,712 (50.8)	1,019 (94.9)	873 (25.9)	348 (73.6)	1,705 (50.5)	101 (7.05)	201 (10.3)	14 (1)	

Supplementary Table 3: Characteristics of individual studies contributing to the current analysis

ED, Emergency department; IQR, interquartile range (25th and 95th percentile)
§ high-risk condition as a defined in the manuscript and only calculated where data were available; * percentage of all patients who received any NAI treatment; ; † number of hospitalised patients in whom data were available; ‡ publications emerging from these datasets whether referring specifically to hospitalization or not
1,530 patients (87.8%) of the total 1,762 included from the Saudi Arabia dataset were recorded as being nationals of Saudi Arabia.

Reference

1. Bantar C, Oliva ME, Ré HA, et al. Severe acute respiratory disease in the setting of an epidemic of swine-origin type A H1N1 influenza at a reference hospital in Entre Ríos, Argentina. Clinical infectious diseases 2009;49:1458-60.
2. Morris SK, Parkin P, Science M, et al. A retrospective cross-sectional study of risk factors and clinical spectrum of children admitted to hospital with pandemic H1N1 influenza as compared to influenza A. BMJ Open 2012;2.
3. Dubnov-Raz G, Somech R, Warschawski Y, Eisenberg G, Bujanover Y. Clinical characteristics of children with 2009 pandemic H1N1 influenza virus infections. Pediatrics International 2011;53:426-30.
4. Lee CK, Lee HK, Loh TP, et al. Comparison of pandemic (H1N1) 2009 and seasonal influenza viral loads, Singapore. Emerging infectious diseases 2011;17:287-91.
5. Gérardin P, El Amrani R, Cyrille B, et al. Low clinical burden of 2009 pandemic influenza A (H1N1) infection during pregnancy on the island of La Reunion. PloS one 2010;5:e10896.
6. Andrews, N., Waight, P.,Yung, C.-F. & Miller, E. 2011. Age-Specific Effectiveness of an Oil-in-Water Adjuvanted Pandemic (H1N1) 2009 Vaccine Against Confirmed Infection in High Risk Groups in England. Journal of Infectious Diseases, 203, 32-39.
